# Gender Differences in Birdshot Chorioretinopathy and the White Dot Syndromes: Do They Exist?

**DOI:** 10.1155/2014/146768

**Published:** 2014-02-09

**Authors:** Lisa J. Faia

**Affiliations:** ^1^Department of Ophthalmology, Oakland University William Beaumont School of Medicine, Rochester, MI 48309, USA; ^2^Retina and Uveitis, Associated Retinal Consultants, P.C., Royal Oak, MI 48073, USA

## Abstract

Inflammatory conditions that affect the posterior pole are diverse. Specifically, birdshot chorioretinopathy and the white dot syndromes present with multiple white dots in the fundus. These diseases appear to affect similar age groups but there is question as to whether or not a difference exists between the genders. This review summarizes the current studies on birdshot chorioretinopathy and the white dot syndromes as they are related to gender, exploring the differences, if any, which may exist between prevalence, clinical presentation, and treatment response for these diseases. Though the specific etiology of these diseases remains unclear, future treatments may be guided as to how these diseases affect the sexes differently.

## 1. Introduction

The spectrum of posterior uveitis disorders is broad. There is a specific group, called the white dot syndromes, which presents with multiple white dots in the fundus. The characteristic inflammatory changes of the choroid and retina are typically yellow-white foci beneath or in the deep retina and include birdshot chorioretinopathy (BCR), acute posterior multifocal placoid pigment epitheliopathy (APMPPE), multiple evanescent white dot syndrome (MEWDS), multifocal choroiditis with panuveitis (MFC), punctate inner choroidopathy (PIC), and acute zonal occult outer retinopathy (AZOOR). These disorders represent a range of presentations, including that of demographics, age, genetic, and gender prevalence. The etiology of these diseases is not completely understood nor is the best approach for treatment of these diseases. The purpose of this review was to assess the current scientific evidence as it is related to the possible gender differences that may exist in birdshot chorioretinopathy and the white dot syndromes.

## 2. Methods and Materials

A systematic review of all the peer-reviewed, English language articles indexed in PubMed about BCR, APMPPE, MEWDS, MFC, PIC, and AZOOR was carried out. For each disease, specific terms were used and reported. Studies with ten or more patients in which patient data included gender and age were reviewed and summarized. Articles were also reviewed for any mention of presentation differences between the sexes and, for those that specified treatment, reviewed to see if differences were noted in the treatment response.

## 3. Results and Discussion

### 3.1. BCR

**Figure 1:**
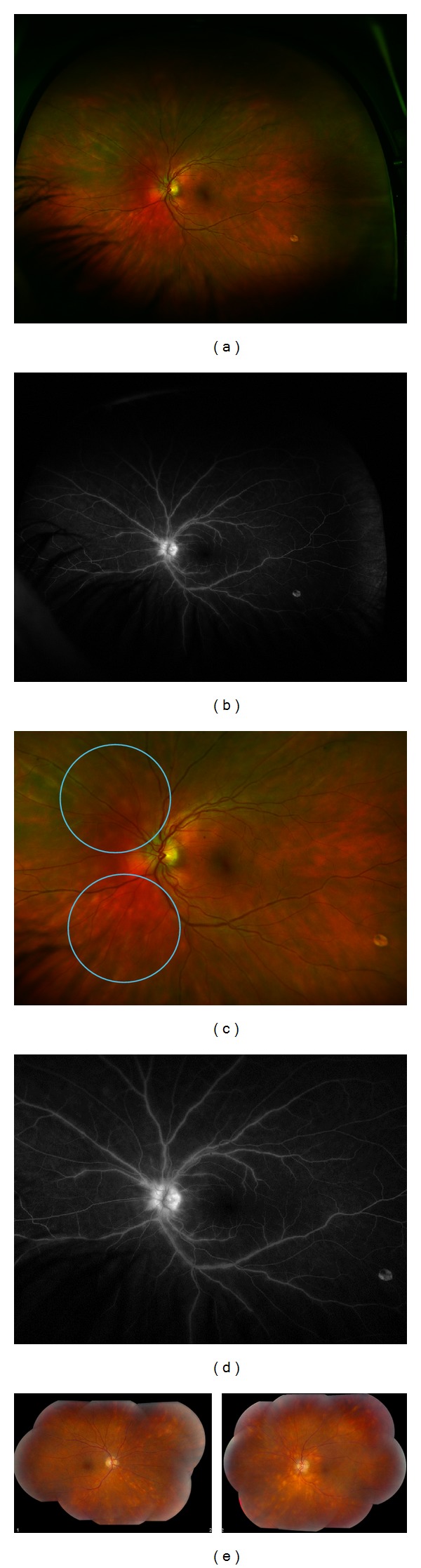
(a) Wide-field fundus photograph of a 34-year-old Caucasian male (diagnosed with BCR one year prior to presentation) with (b) corresponding fluorescein angiogram demonstrating vasculitis. (c) Magnified view of the classic lesions (blue circles) and (d) magnified view of vasculitis and late optic disc leakage. No prior treatment. (e) In contrast, bilateral fundus photography of a 55-year-old Caucasian woman diagnosed with BCR two years prior to presentation with more impressive lesions and vascular sheathing. No prior treatment.

Birdshot chorioretinopathy (BCR) is a rare, chronic, bilateral, posterior inflammatory disease involving the retina and the choroid. The earliest report of this disorder was in 1949 by Franceschaetti and Babel as candle wax spot chorioretinopathy (“la choriorétinite en täche de bougie”) [1]. Ryan and Maumenee coined the term “birdshot retinochoroidopathy” to describe the distinctive lesions seen in the fundus, characterized by multiple, small, white spots that had the appearance of the scatter from a shotgun (Figure 1) [2].

BCR is relatively uncommon, ranging from 1.2 to 7.9% of patients with posterior uveitis [3, 4]. It mostly affects those of Northern European ancestry and those of middle age (average age 48–53), though the range has been reported between 15 and 79 years old [5, 6]. In Shah et al. review, one of the largest reviews on birdshot chorioretinopathy, there was slight female predominance (54.1%), though there have been other studies that have shown a near equal male : female ratio and some with slight male predominance [6–10].

In evaluating the current literature for BCR, data was obtained from 16 articles (Table 1) [6, 8, 9, 11–23]. In PubMed, the term “birdshot chorioretinopathy,” using all or parts of the term, brought up 112 articles. After reviewing and eliminating reports with less than 10 patients, no specificity on patient data for gender and age, and those in which data was repeated, 16 reports remained. Eleven of the 16 reports revealed female predominance, ranging from 54.1 to 100%. When all patients from these articles were considered (*n* = 1157), 669 patients (58%) were female. The mean age was 53.3 years old, with a range from 46.5 to 61 years old.

### 3.2. APMPPE

**Figure 2:**
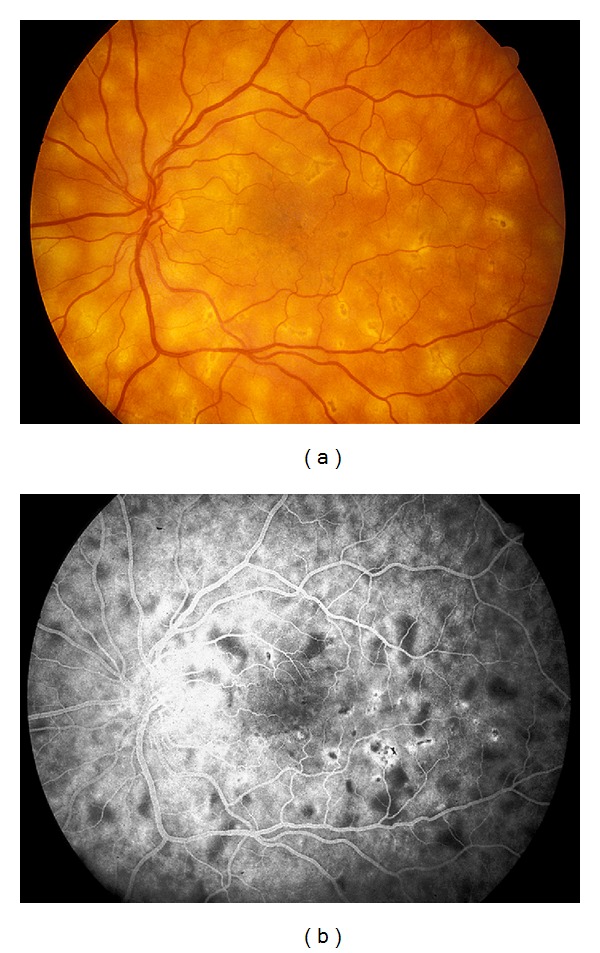
(a) Fundus photograph and corresponding (b) midphase fluorescein angiogram showing blockage of some lesions and the beginning of staining of other lesions as the later phase begins in APMPPE.

APMPPE was first described by Gass in 1968 as a syndrome of multiple, large, placoid lesions at the level of the retinal pigment epithelium that are associated with temporary vision loss [24]. It affects both men and women without preference, usually of good health between the ages of 20 and 50 years old [25]. Vision loss is usually bilateral but may be asymmetric. APMPPE is characterized by bilateral, multifocal yellowish-white placoid lesions usually less than 1 disc diameter in size found in the posterior pole. Classically, these lesions, on fluorescein angiogram, “block early, stain late (Figure 2).” The lesions fade over 1-2 weeks, usually without significant sequelae. Though the etiology is not well understood, it has been postulated that a possible viral agent may be the inciting factor, as patients report a preceding viral prodrome.

In evaluating the current literature for APMPPE, data was obtained from 3 articles (Table 2) [26–28]. In PubMed, the term “acute posterior multifocal placoid pigment epitheliopathy,” using all or parts of the term, brought up 205 articles. After reviewing and eliminating reports with less than 10 patients, no specificity on patient data for gender and age, and those in which data was repeated, 3 reports remained. None of the reports revealed female predominance, ranging from 45.5 to 50%. When all patients from these articles were considered (*n* = 405), 185 patients (46%) were female. The mean of the average age was 27.1 years old, with a range from 26.2 to 28.6 years old.

### 3.3. MEWDS

**Figure 3:**
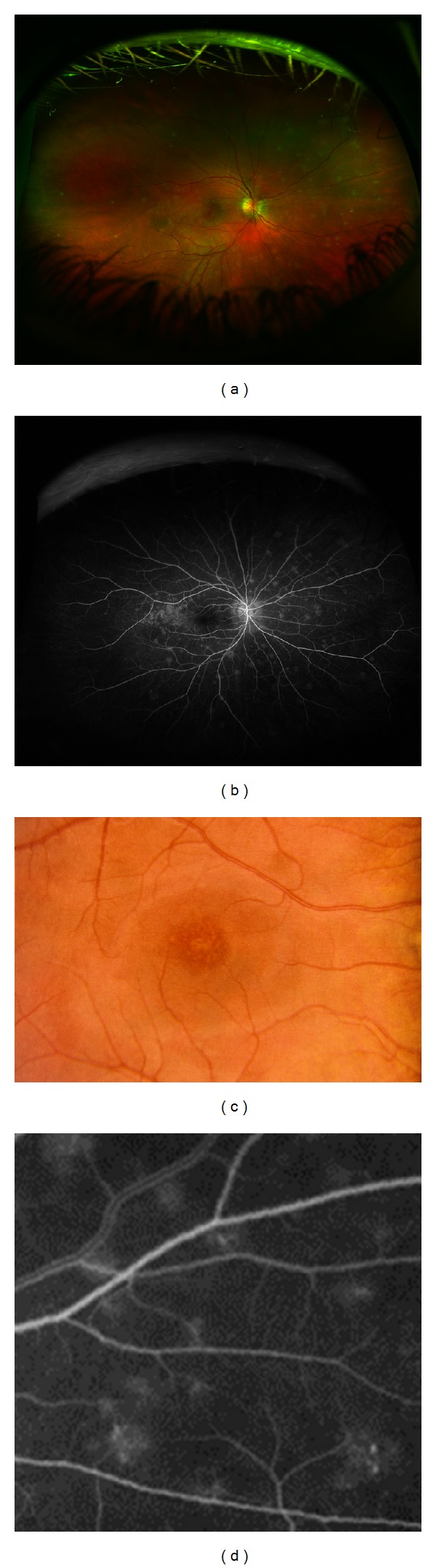
(a) Fundus photograph and corresponding (b) fluorescein angiogram (FA) demonstrating classic wreath-like patterns in MEWDS. (c) Fundus photograph of the macula of different patient demonstration foveal granularity and (d) magnified view of the wreath-like patterns seen on FA in MEWDS.

MEWDS, first described by Jampol et al., presents with numerous small, discrete white lesions in the deep retina or level of the RPE and appears in the posterior pole and extends to the midperiphery [29]. Classically, the fluorescein demonstrates wreath-like lesions and granular appearance to the fovea (Figure 3). Though usually unilateral in young, myopic women ages 20 to 45 years old, there have been bilateral cases described [30]. A preceding viral illness has been reported in approximately 1/3 of cases, and though the cause is unknown, a viral etiology has been suggested. This disease usually resolves spontaneously.

In evaluating the current literature for MEWDS, data was obtained from 3 articles (Table 3) [13, 31, 32]. In PubMed, the term “multifocal evanescent white dot syndrome,” using all or parts of the term, brought up 151 articles. After reviewing and eliminating reports with less than 10 patients, no specificity on patient data for gender and age, and those in which data was repeated, 3 reports remained. Two of the three reports revealed female predominance, ranging from 50 to 91%. When all patients from these articles were considered (*n* = 77), 57 patients (74%) were female. The mean of the average age was 28.7 years old, with a range from 28–29.9 years old.

### 3.4. MFC

**Figure 4:**
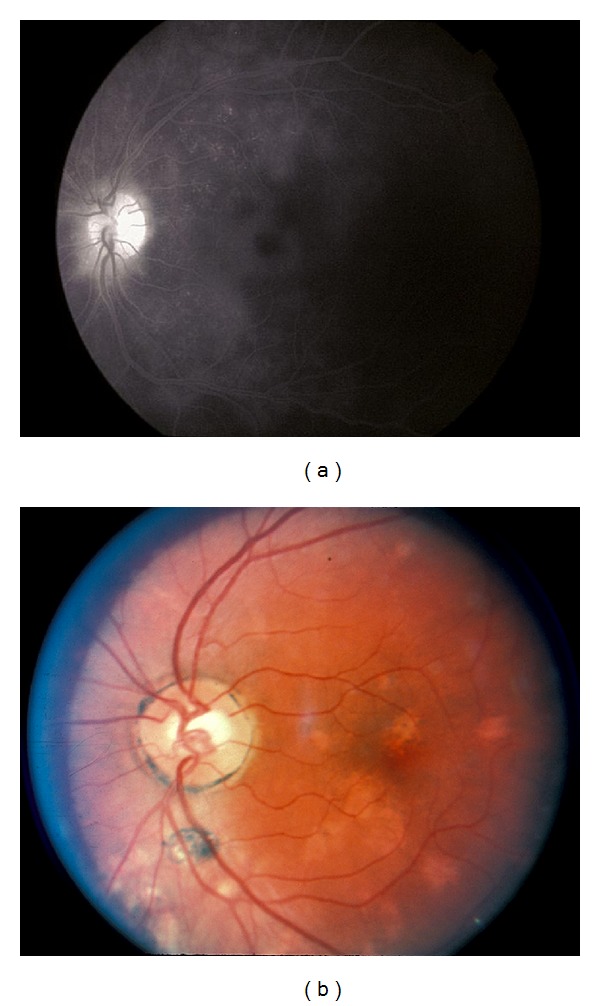
(a) Fluorescein angiogram of a patient with MFC demonstrating concurrent macular edema. (b) Fundus photograph of a patient with MFC requiring systemic immunosuppression.

MFC, unlike classic APMPPE and MEWDS, is more likely to have irreversible visual damage and impairment (Figure 4). This syndrome simulates presumed ocular histoplasmosis (POHS) except that patients present with vitreous cells and inflammation. The punched-out chorioretinal scars with pigmented borders found in the posterior pole and periphery are similar to those in POHS. There is frequent development of choroidal neovascular membranes, which can cause severe vision loss [33]. This disease is usually bilateral with a predilection for patients in their third decade. Though the cause is unknown, it has been hypothesized that an exogenous pathogen may sensitize the individual, with subsequent episodes not requiring the inciting antigen. MFC tends to be a chronic disorder with, generally, a poorer visual prognosis. Some patients require systemic immunosuppression, while other treatments, such as photodynamic therapy and antivascular endothelial growth factor, are used to treat the resultant CNVM [34–36].

In evaluating the current literature for this review for MFC, data was obtained from 22 articles (Table 4) [33–35, 37–56]. In PubMed, the term “multifocal choroiditis and panuveitis,” using all or part of the term, brought up 184 articles. After reviewing and eliminating reports with less than 10 patients, no specificity on patient data for gender and age, and those reports in which data was repeated, 22 articles remained. All reports revealed female predominance, ranging from 55 to 100%. When all patients from these articles were considered (*n* = 538), 406 patients (75%) were female. The mean of the average age was 39.2 years old, with a range from 30.2 to 57 years old.

### 3.5. PIC

**Figure 5:**
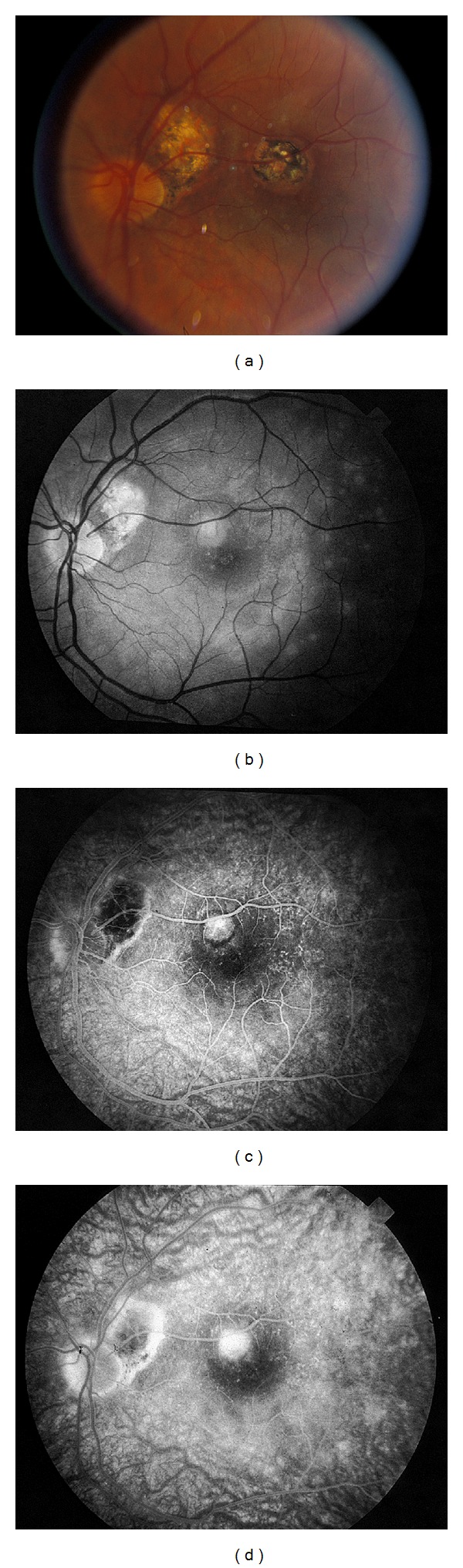
(a) Fundus photography and corresponding fluorescein angiogram ((b)–(d)) of a young woman with PIC demonstrating leakage consistent with a choroidal neovascular membrane.

PIC, a possible variant of MFC, was first described by Watzke et al [57]. This disease was originally described in young, myopic women with punched-out lesions of the posterior pole without ocular inflammation. Like MFC, CNVM may develop and contribute to vision loss (Figure 5).

In evaluating the current literature for PIC, data was obtained from 13 articles (Table 5) [33, 57–64]. In PubMed, the term “punctate inner choroidopathy,” using all or parts of the term, brought up 76 articles. After reviewing and eliminating reports with less than 10 patients, no specificity on patient data for gender and age, and those in which data was repeated, 13 articles remained. All 13 articles revealed female predominance, ranging from 64 to 100%. When all patients from these articles were considered (*n* = 471), 400 patients (85%) were female. The mean of the average age was 33.1 years old, with a range from 26 to 41.5 years old.

### 3.6. AZOOR

AZOOR, thought of predominantly in young women, includes a rapid loss of one or more large zones of outer retinal function and photopsias with minimal fundus changes. Though the cause is unknown, 28% of patients had associated autoimmune diseases, such as Hasimoto's thyroiditis and relapsing transverse myelopathy [65]. No treatment has found to be effective. In Gass' series, 78% of patients with AZOOR had stabilization of the visual field loss and 20% had improvement [65].

In evaluating the current literature for AZOOR, data was obtained from 5 articles (Table 6) [66–70]. In PubMed, the term “acute zonal occult outer retinopathy,” using all or part of the term, brought up 82 articles. After reviewing and eliminating reports with less than 10 patients, no specificity on patient data for gender and age, and those with repeated data, 5 articles remained. All articles revealed female predominance, ranging from 75 to 93%. When all patients from these articles were considered (*n* = 190), 150 patients (79%) were female. The mean of the average age was 38 years old, with a range from 33 to 49.1 years old.

### 3.7. Summary of Gender Differences in Prevalence

A summary of the gathered data from this paper is provided in Table 7. A review of the presented data appears to demonstrate female predominance, in order from most to least, in the following diseases: PIC > AZOOR > MFC > MEWDS. There appeared to be very slight female predominance in BCR in this review. Very slight male predominance was seen in APMPPE in this review. As for age at onset, from youngest to oldest, this review revealed APMPPE > MEWDS > PIC > AZOOR > MFC > BCR. The BCR patients, on average, were twice as old as the patients of the other WDS for age of onset.

### 3.8. Gender Differences in Clinical Presentations

Though some of the above white dot syndromes have differences in the ratios of involvement of men to women, no clinical differences have been described between the sexes [1–3, 5, 8–15, 17–20, 24, 26–29, 31–33, 37, 38, 44–47, 50–52, 54–59, 61, 62, 65, 68–71]. In review of the reports, no distinctions were made between the genders in age of onset, initial clinical findings, or severity of disease.

### 3.9. Gender Differences in Treatment and Prognosis

Though this review revealed female predominance in PIC, AZOOR, MFC, and MEWDS, treatment differences have not been documented between these and the other white dot syndromes [34, 35, 48, 61]. This should be considered in future studies as differences in response to steroids in SLE, another female predominant autoimmune disease (9 : 1), have been noted [72]. Estrogens have been implicated as enhancers of the immune system (with androgens and progesterone being considered immunosuppressors), and such possible explanations have been given for the differences in steroid response in SLE [72]. The other WDS diseases, without preference or with male predominance, also have not had treatment differences noted in the literature [5, 7, 21–23, 71, 73, 74]. BCR, MFC, and PIC in general have poorer VA prognoses than APMPPE and MEWDS, though systemic immunomodulatory therapy may help to decrease the amount of vision loss in BCR, MFC, and PIC. MFC and MEWDS appear to be female dominant and on the different ends of the spectrum for disease prognosis, suggesting that gender, for these diseases, may have little to no effect on visual prognosis. The most abundant amount of data in the literature on treatment of the white dot syndrome diseases concerns BCR. Articles on the use of intravitreal triamcinolone, intravenous immunoglobulin, cyclosporine alone, cyclosporine plus mycophenolate mofetil, methotrexate, infliximab, and daclizumab can be found in the literature on the treatment of BCR with varying success and no mention of response differences between the genders [5, 7, 21–23, 71, 73, 74].

### 3.10. A Hormonal Difference?

Sex hormones influence the immune system, resulting in females having higher immunoglobulin levels and mounting stronger immune responses following immunizations or infections than males [75]. However, this also increases woman's susceptibility to autoimmune diseases [75]. Abnormal hormone levels may trigger disease [75]. BCR tends to involve older patients, including women who may be menopausal. This is less likely to occur in the other WDS diseases, as they tend to be younger. This may be a possible reason why the ratio is much closer in female : male involvement in BCR than such diseases as PIC, AZOOR, MFC, and MEWDS. Unfortunately, this does not explain the near equal development of APMPPE in males and females, as the patients tend to be younger and this disease usually follows a viral prodrome. Clearly, there is something more. Other factors, such as the involvement of HLA A29 factor in BCR, may influence the occurrence of disease [71].

## 4. Conclusion

In conclusion, though in this review PIC, AZOOR, MFC, and MEWD were found to have female predominance, there does not appear to be a significant difference in clinical presentation nor in the treatment of these diseases between the genders. BCR and APMPPE appear to affect both men and women equally and again, both in presentation and treatment, there does not appear to be a significant difference between the genders. Though estrogens have been implicated in the manipulations of the immune system, further work is needed to truly elicit how estrogen levels may affect prevalence, presentation, and treatment in these ocular diseases.

## Figures and Tables

**Table 1:** Reports for birdshot chorioretinopathy.

Author	Year published	No. of patients	No. of women (%)	Average age (years)
Keane et al. [11]	2013	12	5 (42)	59
Yang and Foster [12]	2013	17	8 (47)	52
Cervantes-Castaneda et al. [23]	2013	49	28 (57)	48.8
Papadia and Herbort [13]	2013	25	19 (76)	49.6
Artornsombudh et al. [22]	2013	22	17 (77.3)	53
Kuiper et al. [14]	2011	16	15 (94)	61
Rothova et al. [21]	2011	76	49 (64)	54
Giuliari et al. [15]	2010	15	15 (100)	52.3
Pagnoux et al. [16]	2010	118	73 (62)	51.5
Trinh et al. [17]	2009	10	4 (40)	46.5
Kappel et al. [18]	2009	63	38 (60)	60.9
Thorne et al. [9]	2008	55	25 (45)	56
Monnet et al. [19]	2006	80	51 (64)	55.6
Shah et al. [6]	2005	522	283 (54.1)	53
Sobrin et al. [20]	2005	23	13 (56.5)	49
Rothova et al. [8]	2004	54	26 (48)	53

**Table 2:** Reports for acute posterior multifocal placoid pigment epitheliopathy.

Author	Year published	No. of patients	No. of women (%)	average Age (years)
Thomas et al. [26]	2012	18	9 (50)	28.6
Fiore et al. [27]	2009	187	85 (45.5)	26.2
Jones [28]	1995	200	91 (45.5)	26.5

**Table 3:** Reports for multiple evanescent white dot syndrome.

Author	Year	No. of patients	No. of women (%)	average Age (years)
Asano et al. [31]	2004	50	39 (78)	29.9
Reddy et al. [32]	1996	16	8 (50)	28.1
Jampol et al. [29]	1984	11	10 (91)	28

**Table 4:** Reports for multifocal choroiditis and panuveitis.

Author	Year	No. of patients	No. of women (%)	average Age (years)
Fung et al. [37]	2013	41	29 (70.7)	38.4
Spaide et al. [38]	2013	17	13 (78.3)	33
Parodi et al. [39]	2013	14	9 (64)	48
Mansour et al. [40]	2012	12	9 (75)	37.8
Atan et al. [41]	2011	30	20 (67)	57
Parodi et al. [42]	2010	27	18 (67)	39
Kotsolis et al. [43]	2010	17	14 (82)	42.7
Haen and Spaide [44]	2008	18	15 (83)	43.2
Kedhar et al. [45]	2007	66	50 (75.8)	49
Thorne et al. [46]	2006	66	50 (76)	45
MacLaren and Lightman [47]	2006	20	11 (55)	37.1
Vianna et al. [48]	2006	19	13 (68)	46.2
Parodi et al. [49]	2004	13	11 (85)	47
Michel et al. [34]	2002	19	15 (79)	34.8
Spaide et al. [35]	2002	17	15 (88)	34.2
Parnell et al. [50]	2001	25	23 (92)	31.1
Vadalà et al. [51]	2001	13	13 (100)	33
Slakter et al. [52]	1997	14	8 (57)	31
Brown Jr. et al. [33]	1996	41	32 (78)	36
Tiedeman [53]	1987	10	6 (60)	36.6
Morgan and Schatz [54]	1986	11	11 (100)	30.2
Dreyer and Gass [55]	1984	28	21 (75)	33
*Watzke and Claussen [56]	1981	40	N/A	N/A

*Not included in data analysis.

**Table 5:** Reports for punctate inner choroidopathy.

Author	Year published	No. of patients	No. of women (%)	average Age (years)
Zhang et al. [58]	2013	42	27 (64)	26
Spaide et al. [38]	2013	13	12 (92)	38
Mansour et al. [40]	2012	24	19 (79)	41.5
Zhang et al. [60]	2012	12	11 (92)	32.9
Zhang et al. [59]	2011	75	54 (72)	32
Patel et al. [61]	2011	12	11 (92)	32
Atan et al. [41]	2011	31	26 (84)	40
Essex et al. [62]	2010	136	126 (93)	32
Menezo et al. [63]	2010	10	8 (80)	40.7
Kedhar et al. [45]	2007	13	12 (92)	29
Gerstenblith et al. [64]	2007	77	69 (90)	30
Brown Jr. et al. [33]	1996	16	15 (94)	30
Watzke et al. [57]	1984	10	10 (100)	26.8

**Table 6:** Reports for acute zonal occult outer retinopathy.

Author	Year published	No. of patients	No. of women (%)	average Age (years)
Jiang et al. [66]	2013	14	13 (93)	33
Saito et al. [67]	2013	11	10 (91)	35
Monson and Smith [68]	2011	130	99 (76)	36.7
Fujiwara et al. [69]	2010	11	10 (91)	49.1
Jacobson et al. [70]	1995	24	18 (75)	35

**Table 7:** Summary of gathered data.

Disease	Average age (years)	Gender analysis (% women)
BCR	53.5	F > M (58%)
APMPPE	27.1	M > F (46%)
MEWDS	28.7	F > M (74%)
MFC	39.2	F > M (75%)
PIC	33.1	F > M (85%)
AZOOR	38	F > M (79%)

BCR: birdshot chorioretinopathy, APMPPE: acute posterior multifocal placoid pigment epitheliopathy, MEWDS: multiple evanescent white dot syndrome, MFC: multifocal choroiditis and panuveitis, PIC: punctate inner choroidopathy, AZOOR: acute zonal occult outer retinopathy.
